# Low-dose lithium against dementia

**DOI:** 10.1186/s40345-020-00188-z

**Published:** 2020-08-03

**Authors:** Christopher Baethge

**Affiliations:** Department of Psychiatry and Psychotherapy, Faculty of Medicine, University of Cologne Medical School, Kerpener Str. 62, 50931 Cologne, Germany

The *International Journal of Bipolar Disorder* has done lithium patients and their doctors a great favor in publishing a concise and practical guide to lithium’s use (Tondo et al. 2019), written by Leonardo Tondo and his co-authors from the International Group for Study of Lithium Treated Patients (IGSLi). Starting from a scholarly sketch of the history of lithium’s discovery and use in psychiatry, they provide a brief overview on its efficacy and predictors of treatment success, give practical advice on initiating lithium (where they may place too much weight on the kindling hypothesis) and on monitoring long-term treatment as well as its most important side effects. They also delve into special topics, such as lithium in pregnancy and lithium’s anti-suicidal properties. All in all a very compelling lithium primer by some of the best-known experts in the field. However, one topic is missing: lithium’s possible neuroprotective effects—a topic that has been revived lately.

A couple of months ago, apparently almost unbeknownst to many experts and largely unnoticed in the public, a paper was published that may fundamentally change dementia care (Forlenza et al. 2019). In the article, Forlenza and his co-workers from Sao Paulo describe a double-blind, placebo-controlled trial of low-dose lithium among patients with mild cognitive impairment (MCI) aimed at slowing cognitive decline and ultimately at preventing progression to dementia.

After 2 years, while cognition remained stable in lithium treated patients, as evidenced by common cognition scales, patients receiving placebo exhibited continuous decline. In an open-label extension phase, 4 years into the study, five out of 31 subjects in the lithium group and 9 out of 30 under placebo had progressed to dementia.

Cutting in half the risk of transition from MCI to dementia? Why didn’t we find such a striking result in the pages of the *New England Journal of Medicine*? Enthusiasm may have been stifled for a number of reasons: For example, the study took many years to be published when the extension phase must have been completed in 2011. With 61 instead of 80, the trial did not reach its planned sample size, and some may have wondered how blinding could have been maintained in a lithium study. What’s more, the placebo sample was slightly older (74.4 versus 71.2 years), and the stated primary outcome shifted between retrospective trial registration in 2010 (Disease-modifying Properties of Lithium in the Neurobiology of Alzheimer’s Disease—Full Text View—ClinicalTrials.gov: accessed September 15 2019), a first publication in 2011 (Forlenza et al. 2011), and the final paper just last spring (Forlenza et al. 2019). Also, from the numbers in the results table, it is difficult to reconcile some of the p-values with re-calculated t-tests, and statistical significance would suffer were tests adjusted for multiplicity. Finally, the difference in switch rates to dementia, arguably the most important outcome, missed statistical the significance by a hair (p = 0.06).

All of this may have raised some eyebrows. Plus, even though some have hypothesized that lithium may be neuroprotective in very low concentrations, as found in drinking water (Kessing et al. 2017), earlier attempts at detecting a signal of cognition preserving properties of lithium in observational studies have produced mixed results (Pfennig et al. 2014; Gerhard et al. 2015). Therefore, if this were a run-of-the-mill antidepressant trial, chances are it would have been discarded, in particular against the backdrop of literally hundreds of trials accrued in decades and in the midst of a wide array of treatment options.

As far as I can tell, however, the study by Forlenza and co-workers is the first low-dose lithium (0.25–0.5 mmol/l) trial in MCI, and it is certainly a remarkable feat to conduct such a study over 4 years. This is why it is justified not to dismiss this work. In taking a closer look it appears the effect sizes are quite strong: A relative risk of 0.54 [95%-CI 0.20–1.42] of transitioning to dementia after 4 years, albeit under the assumption that none of the drop-outs became demented. Provided all drop-outs would have had dementia plus those already diagnosed in the study, the relative risk would have risen to 0.76 [0.53–1.08], still a sizeable effect.

On the level of scales, namely the Clinical Dementia Rating Scale and the Alzheimer’s Disease Assessment Scale, Hedges’ g stands at 0.5 and 0.6, respectively, an effect in the medium range. However, the effect is larger than what we know for many other psychiatric indications (e.g., (Henssler et al. 2018)) and certainly what is available in the dementia field. For comparison: in a meta-analysis on cognitive training in MCI, Hill and co-workers found a lower overall effect of 0.38 [0.14–0.62] (Hill et al. 2017).

If there was an abundance of effective treatments for dementia, I would probably be more reserved regarding the signal published by Forlenza et al., but we all know that patients with dementia and their relatives are desperately waiting for some drug or treatment helping them in their suffering. It is an interesting thought experiment for psychiatrists to ask themselves whether they, after having received a diagnosis of MCI, would be willing to give low-dose lithium a try—it is conceivable that many would.

Taken together, in a disorder where we can offer so little to patients, the effect sizes presented by Forlenza et al. are substantial, encouraging and a rare silver lining. Given the uncertainties in this study and the fact that effect sizes can be larger in early and small studies, we do not know whether the results will hold, and we probably won’t know until 2022 when a very similar study in Pittsburgh is scheduled to end (Lithium As a Treatment to Prevent Impairment of Cognition in Elders–Full Text View–ClinicalTrials.gov: Accessed Sept 17 2019). Until then, let us all hope that lithium, this remarkable alkali metal, will turn out to be crucial not only for patients with bipolar disorder and for batteries, and that future primers of lithium treatment will contain a recommendation to use lithium salts in the treatment of MCI.

## Data Availability

Not applicable.
